# Associations of Sociodemographic Factors, Lifestyle Habits, and Insomnia Severity with Obesity Indices in Spanish Workers: Sex-Specific Differences

**DOI:** 10.3390/medsci13040271

**Published:** 2025-11-14

**Authors:** José Luis Ribes Valles, Pedro Juan Tárraga López, Ángel Arturo López González, Irene Coll Campayo, Carla Busquets-Cortés, José Ignacio Ramírez-Manent

**Affiliations:** 1 Son Llatzer Hospital, 07198 Palma, Spain; 2 Faculty of Medicine, University of Castilla La Mancha (UCLM), 02008 Albacete, Spain; 3 ADEMA-Health Group of IUNICS, 07009 Palma, Spain; 4 Balearic Islands Health Service, 07010 Palma, Spain; 5 Faculty of Medicine, University of the Balearic Islands, 07120 Palma, Spain

**Keywords:** obesity, insomnia, sleep disorders, life style, sociodemographic variables, CUN BAE

## Abstract

**Background:** Obesity and insomnia are prevalent public health issues with shared behavioral and physiological pathways. However, their interplay remains understudied in occupational cohorts. Obesity and insomnia are prevalent public health issues with shared behavioral and physiological pathways. However, their interplay remains understudied in occupational cohorts. This study aimed to evaluate the associations of sociodemographic factors, lifestyle habits, and insomnia severity with multiple obesity indices in a large population of Spanish workers. **Methods:** We conducted a cross-sectional analysis of 84,898 workers (2021–2024). Data were obtained from annual occupational health assessments conducted across multiple Spanish regions between 2020 and 2024. Insomnia severity was assessed using the Insomnia Severity Index (ISI), dietary quality using the 14-item Mediterranean Diet Adherence Screener (MEDAS-14), and physical activity using the International Physical Activity Questionnaire—Short Form (IPAQ-SF). Adiposity was measured using Body Mass Index (BMI), waist-to-height ratio (WtHR), the Clínica Universidad de Navarra–Body Adiposity Estimator (CUN-BAE), and the Metabolic Score for Visceral Fat (METS-VF). Multivariable logistic regression models were used to examine associations adjusted for age, sex, education, and occupational social class. **Results:** Higher ISI scores were significantly and independently associated with elevated adiposity across all indices, with the strongest association observed for METS-VF (odds ratio = 1.19, 95% CI 1.14–1.25, *p* < 0.001). Women showed higher mean CUN-BAE and METS-VF values than men (CUN-BAE: 37.4 ± 6.2 vs. 25.6 ± 6.4; *p* < 0.001; METS-VF: 5.7 ± 0.7 vs. 6.4 ± 0.6; *p* < 0.001), despite lower BMI (25.3 ± 4.8 vs. 26.8 ± 4.3; *p* < 0.001). Lower physical activity (OR = 5.70; 95% CI 4.91–6.50), poor adherence to the Mediterranean diet (OR = 3.29; 95% CI 2.88–3.70), smoking (OR = 1.29; 95% CI 1.22–1.36), and lower occupational class (Class III: OR = 1.77; 95% CI 1.56–1.97) were also significantly associated with higher obesity markers. Associations were more pronounced among women and participants with severe insomnia symptoms. **Conclusions:** Insomnia severity, sociodemographic disadvantage, and unhealthy behaviors (low physical activity, poor diet, smoking) were all independent correlates of general and visceral adiposity. The findings underscore the need for comprehensive workplace health programs that integrate sleep quality assessment, dietary improvement, and physical activity promotion to prevent obesity and its metabolic consequences.

## 1. Introduction

Obesity is a multifactorial and chronic condition characterized by excessive or abnormal accumulation of body fat that impairs health and increases the risk of numerous non-communicable diseases. Over the past decades, the global prevalence of obesity has reached pandemic levels, affecting more than 1 billion individuals worldwide [1,2] and contributing substantially to the burden of cardiovascular disease, type 2 diabetes mellitus (T2DM), certain cancers, musculoskeletal disorders, and premature mortality [3,4,5,6,7,8]. In Spain, recent estimates indicate that over 21% of adults meet the criteria for obesity, with an upward trend particularly evident in working-age populations [9,10].

From a pathophysiological perspective, obesity is associated with complex disturbances in neuroendocrine regulation, adipokine signaling, energy balance, and inflammatory pathways. Visceral adipose tissue, in particular, functions as a metabolically active endocrine organ that secretes cytokines and adipokines such as interleukin-6 (IL-6), tumor necrosis factor-alpha (TNF-α), leptin, and adiponectin, contributing to systemic inflammation, insulin resistance, and endothelial dysfunction [11,12,13,14,15,16]. These mechanisms promote the development of cardiometabolic conditions, including hypertension, dyslipidemia, non-alcoholic fatty liver disease (NAFLD), and atherosclerosis [17,18,19,20].

Traditionally, the body mass index (BMI) has been used as a standard tool to define obesity; however, it presents significant limitations. Despite extensive evidence linking individual behaviors to obesity, few large-scale occupational studies have simultaneously evaluated sociodemographic, behavioral, and sleep-related factors, leaving a gap in understanding how these determinants interact to influence obesity risk. BMI does not account for differences in fat distribution or distinguish between lean and fat mass, leading to diagnostic inaccuracies, especially in individuals with sarcopenic obesity or normal-weight central adiposity [21]. Consequently, alternative anthropometric and metabolic indices have been proposed to more accurately reflect body fat composition and cardiometabolic risk. Among these, the waist-to-height ratio (WtHR) offers a simple yet powerful measure of central obesity, with a cut-off value of 0.5 serving as a reliable predictor of metabolic dysfunction [22].

Given that each anthropometric index has inherent limitations, several complementary measures were included to improve the robustness of the analyses [23,24,25,26,27,28,29,30].

While lifestyle factors such as diet quality, physical activity, and smoking habits are well-established contributors to obesity, emerging evidence also points to the role of sleep health—particularly insomnia—in shaping adiposity patterns and metabolic outcomes. Insomnia is a prevalent sleep disorder characterized by difficulty initiating or maintaining sleep, or experiencing non-restorative sleep, despite adequate opportunity and circumstances for rest [31]. Chronic insomnia is associated with significant impairments in daytime functioning, mood disturbances, and reduced quality of life [32,33,34].

Epidemiological studies report that 10% to 30% of adults experience clinically significant insomnia, with higher prevalence among women, older individuals, and those in high-stress occupations [35]. The pathophysiology of insomnia involves dysregulation of the hypothalamic–pituitary–adrenal (HPA) axis, increased sympathetic activity, and alterations in sleep–wake homeostasis, all of which may affect appetite regulation, glucose metabolism, and fat storage [36,37,38]. Sleep restriction and poor sleep quality have been shown to impair leptin and ghrelin balance, increase cortisol levels, and elevate proinflammatory cytokines, thereby promoting weight gain and central fat accumulation [39,40,41].

Several population-based studies have demonstrated associations between insomnia and increased risk of obesity, metabolic syndrome, hypertension, and T2DM, even after adjusting for traditional risk factors [42,43,44]. However, few large-scale investigations have comprehensively examined how insomnia severity relates to validated adiposity indices, particularly within working populations exposed to high stress, sedentary behavior, and irregular schedules.

Accordingly, the present study aims to assess the associations between sociodemographic factors, lifestyle habits (diet, smoking, physical activity), and insomnia severity with multiple obesity indices in a large population of Spanish workers. By integrating anthropometric, metabolic, behavioral, and sleep-related variables, this study seeks to contribute new insights into the multidimensional determinants of obesity and support the development of more targeted and holistic workplace health interventions.

A secondary objective was to explore sex-specific differences in the associations among sociodemographic, behavioral, and obesity-related factors.

## 2. Methods

### 2.1. Study Design and Population

**Figure 1:**
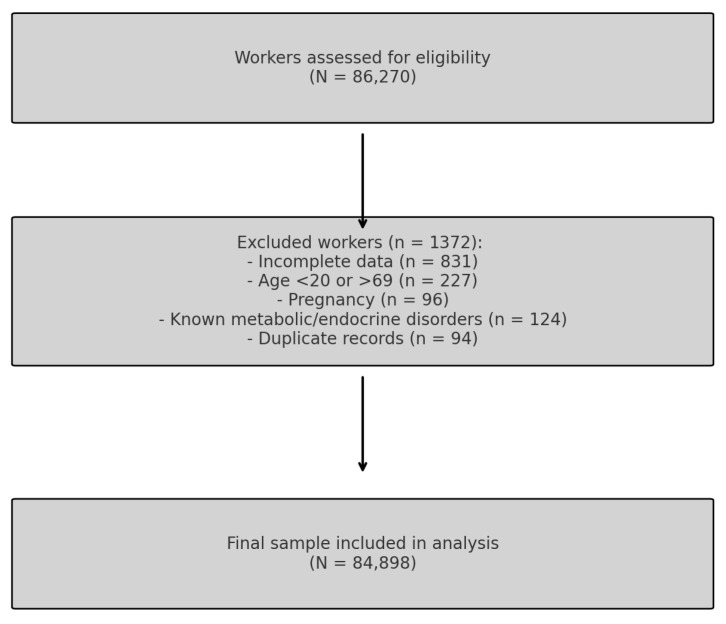
Participant Flow Diagram.

This cross-sectional observational study was carried out as part of a nationwide occupational health surveillance program in Spain. Data were collected between January 2021 and December 2024 during routine medical evaluations conducted at certified occupational health centers (Figure 1). Health surveillance was performed by Quirónprevención, a national occupational health provider in Spain. Participants were recruited from the industrial, commercial, and service sectors through company-based occupational health programs. They were drawn from various occupational health services across several Spanish Autonomous Communities that voluntarily agreed to participate in the study.

All data were anonymized before analysis to ensure participant confidentiality. The study was approved by the Institutional Research Ethics Committee and complied with the ethical principles outlined in the Declaration of Helsinki. All participants provided written informed consent prior to their inclusion in the study.

### 2.2. Eligibility Criteria

To ensure internal validity and generalizability, strict inclusion and exclusion criteria were applied.

Inclusion criteria:

- Age between 18 and 69 years at the time of assessment, to represent the active working population.
- Employment as an active worker in any economic sector with a registered occupational record
- Availability of complete and validated data for all variables of interest, including anthropometric, biochemical, lifestyle, sociodemographic, and sleep-related measures

Exclusion criteria:

- Incomplete or missing data essential for the calculation of obesity indices or insomnia severity
- Age < 18 or >69 years
- Pregnancy at the time of examination
- Diagnosed metabolic or endocrine disorders (e.g., uncontrolled diabetes, thyroid dysfunction, malignancy, advanced chronic kidney disease)
- Duplicate records or inconsistencies in key demographic identifiers

From an initial pool of 86,270 individuals, 1372 workers were excluded based on these criteria, yielding a final sample of 84,898 participants eligible for analysis (Figure 1).

### 2.3. Data Collection and Variable Definitions

Anthropometric and clinical parameters were measured by trained personnel using standardized techniques and calibrated instruments. Height and weight were measured with participants in light clothing and without shoes. Waist circumference was measured at the midpoint between the lower rib and the iliac crest; hip circumference at the widest part of the buttocks. Systolic and diastolic blood pressure were measured in a seated position after at least five minutes of rest. Blood samples were drawn after a 10–12 h overnight fast to assess fasting plasma glucose, triglycerides, total cholesterol, HDL-c, and LDL-c, which were analyzed in ISO-certified laboratories.

Sociodemographic variables included sex, age, and occupational social class, the latter determined based on the Spanish National Classification of Economic Activities (CNAE-11). Social class was categorized as Class I (managers, professionals), Class II (intermediate occupations), or Class III (manual and routine occupations), following the guidelines of the Spanish Society of Epidemiology [45].

#### 2.3.1. Assessment of Lifestyle Factors

Health-related lifestyle habits were self-reported using validated tools.

- Dietary quality was evaluated through the 14-item Mediterranean Diet Adherence Screener (MEDAS-14) [46]. A score ≥ 9 indicated adequate adherence to the Mediterranean dietary pattern, which emphasizes the consumption of fruits, vegetables, whole grains, legumes, olive oil, and moderate intake of fish and red wine [47].
- Physical activity was assessed using the International Physical Activity Questionnaire—Short Form (IPAQ-SF), a validated instrument used to estimate activity levels in population studies. Participants were classified as physically active or inactive according to standardized scoring protocols [48,49].
- Smoking status was classified as current smoker or non-smoker based on self-report.

#### 2.3.2. Evaluation of Insomnia Severity

Sleep disturbances were assessed using the Insomnia Severity Index (ISI), a 7-item self-administered questionnaire that evaluates difficulties in sleep initiation, sleep maintenance, early morning awakenings, sleep dissatisfaction, interference with daily functioning, noticeability of sleep problems, and distress caused by poor sleep. The Insomnia Severity Index (ISI) is a validated and widely used tool in both clinical and research settings. It evaluates perceived insomnia symptoms over the past two weeks through seven items addressing sleep onset latency, sleep maintenance, satisfaction, interference with daily functioning, and distress [50]. The ISI has demonstrated strong psychometric properties and clinical utility for grading insomnia severity in occupational health studies.

Insomnia severity was analyzed primarily as a categorical variable, classified into four levels according to established cutoffs [51]:

- No insomnia (0–7)
- Subthreshold insomnia (8–14)
- Moderate insomnia (15–21)
- Severe insomnia (22–28)

The ISI is a widely validated instrument in both clinical and occupational populations and has shown high internal consistency and test–retest reliability.

#### 2.3.3. Obesity and Adiposity Indices

A multidimensional approach was used to evaluate adiposity through four validated indices, providing a more comprehensive understanding of body fat distribution and metabolic risk.

While BMI is the most commonly used measure, it does not distinguish between fat and lean mass.

The waist-to-height ratio (WtHR) offers a simple yet effective proxy for central adiposity, while the Clínica Universidad de Navarra–Body Adiposity Estimator (CUN-BAE) estimates total body fat percentage by accounting for age and sex.

The Metabolic Score for Visceral Fat (METS-VF) is a novel index that integrates sex, age, WtHR, fasting glucose, and triglycerides to estimate visceral adiposity and its associated metabolic dysfunction.

The four indices were defined and calculated as follows:

1. Body Mass Index (BMI): calculated as weight in kilograms divided by height in meters squared (kg/m^2^); obesity defined as BMI ≥ 30 kg/m^2^.
2. Waist-to-Height Ratio (WtHR): waist circumference (cm) divided by height (cm); central obesity defined as WtHR ≥ 0.50.
3. Clínica Universidad de Navarra–Body Adiposity Estimator (CUN-BAE): a regression-based formula incorporating age, sex, and BMI to estimate body fat percentage; obesity defined as CUN-BAE ≥ 35% [52].
4. Metabolic Score for Visceral Fat (METS-VF): a composite index that integrates sex, age, WtHR, fasting glucose, and triglycerides; a score ≥ 6.3 was used to define high visceral adiposity [53].

**Figure 2:**
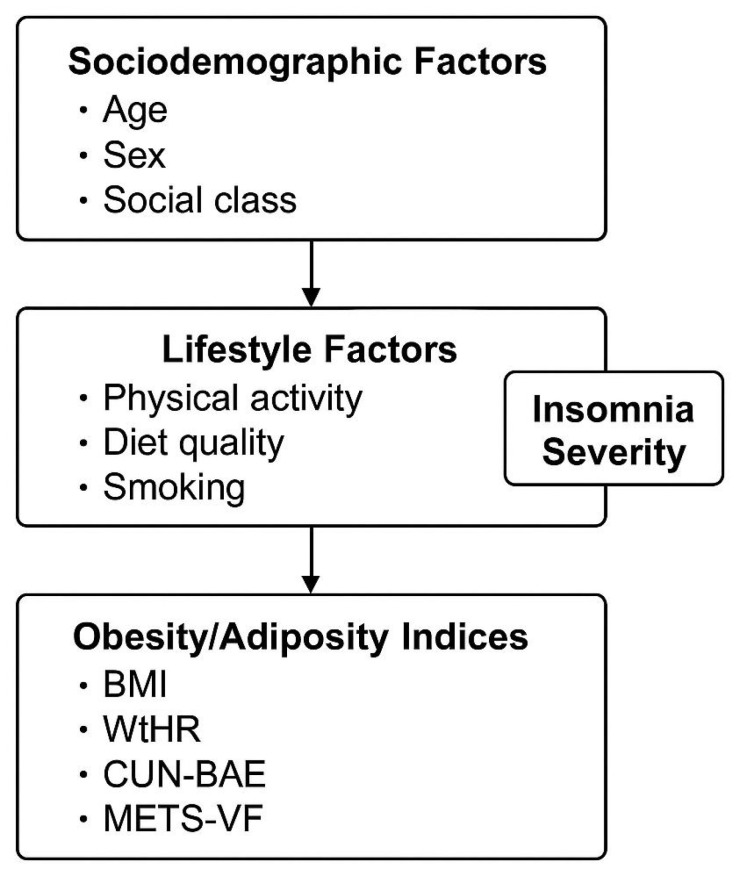
Conceptual framework illustrating the unidirectional associations between sociodemographic factors, lifestyle factors (physical activity, diet quality, and smoking), and insomnia severity (independent variables) with obesity/adiposity indices (BMI, WtHR, CUN-BAE, and METS-VF). Arrows represent hypothesized directional associations, not causal relationships.

These indices were selected based on prior validation in Mediterranean and working populations and offer complementary perspectives on adiposity distribution and metabolic risk (Figure 2).

### 2.4. Statistical Analysis

Descriptive statistics were used to summarize the characteristics of the population. Continuous variables were reported as means and standard deviations (SD), and categorical variables as frequencies and percentages. Comparisons between groups were made using Student’s *t*-test or ANOVA for continuous variables and the chi-square test for categorical variables.

To examine the independent associations between sociodemographic characteristics, health behaviors, insomnia severity, and the presence of obesity, a series of multivariate logistic regression models were fitted for each adiposity index. Between-group analyses were performed to compare men and women with respect to sociodemographic, lifestyle, and clinical variables using Student’s *t*-tests for continuous data and chi-square tests for categorical data. Subsequently, multivariable logistic regression models were fitted for each adiposity index as the dependent variable, with sociodemographic characteristics, lifestyle factors, and insomnia severity entered as independent predictors. Sensitivity analyses were performed by sex. Results were reported as odds ratios (ORs) with 95% confidence intervals (CIs). Statistical significance was defined as *p* < 0.05. All analyses were conducted using IBM SPSS Statistics for Windows, version 29.0 (IBM Corp., Armonk, NY, USA).

## 3. Results

Table 1 provides a comprehensive overview of the sociodemographic, anthropometric, clinical, and lifestyle characteristics of the study population, stratified by sex. Significant sex-based differences were observed across all variables (*p* < 0.001). Standard deviations were reported to describe population variability rather than precision of the mean, as recommended for large epidemiological datasets. Men presented higher mean values for weight, waist circumference, blood pressure, triglycerides, LDL-c, and glucose, while women showed higher HDL-c levels and healthier lifestyle behaviors, such as adherence to the Mediterranean diet and physical activity. Notably, insomnia severity differed markedly by sex, with women more frequently reporting no insomnia (64.8%) and men showing a higher prevalence of moderate to severe insomnia. These findings highlight relevant sex-specific differences in cardiometabolic risk profiles and sleep disturbances in occupational cohorts.

Table 2 presents the distribution of BMI, waist-to-height ratio (WtHR), CUN-BAE, and METS-VF across categories of age, social class, smoking status, dietary habits, physical activity, and insomnia severity. A consistent trend of increasing adiposity was observed with age and lower socioeconomic status in both sexes. Physical inactivity and poor adherence to the Mediterranean diet were associated with markedly higher values in all obesity indices. Importantly, there was a stepwise increase in obesity index scores across insomnia severity categories, with the highest values among those with severe insomnia. These findings suggest a cumulative burden of sociodemographic disadvantages, unhealthy behaviors, and sleep disturbances on obesity and visceral fat accumulation.

Table 3 reports the prevalence of obesity and high adiposity across four indices (BMI, WtHR, CUN-BAE, METS-VF), stratified by relevant sociodemographic and behavioral factors. The data reveal that the proportion of individuals with elevated adiposity rises with age, lower social class, smoking, poor diet, and physical inactivity. Importantly, a clear dose–response relationship is evident between insomnia severity and all obesity metrics. For example, severe insomnia is associated with markedly higher obesity prevalence (e.g., 39.8% for BMI in men, 37.2% in women). This table underscores the need to integrate sleep health into comprehensive strategies addressing obesity and metabolic risk in the workplace.

Table 4 displays the adjusted odds ratios (ORs) and 95% confidence intervals for having obesity or high visceral adiposity based on four indices, considering sex, age group, social class, smoking status, diet, physical activity, and insomnia severity. Male sex, older age, lower social class, smoking, unhealthy diet, and physical inactivity were significantly associated with increased odds of obesity across all indices. Insomnia severity showed a strong and independent association with obesity risk: compared to those without insomnia, individuals with moderate or severe insomnia had substantially higher ORs for all outcomes, with the strongest associations observed for METS-VF. These results highlight insomnia as a potential modifiable risk factor for visceral obesity and support the inclusion of sleep assessments in occupational health programs.

**Figure 3:**
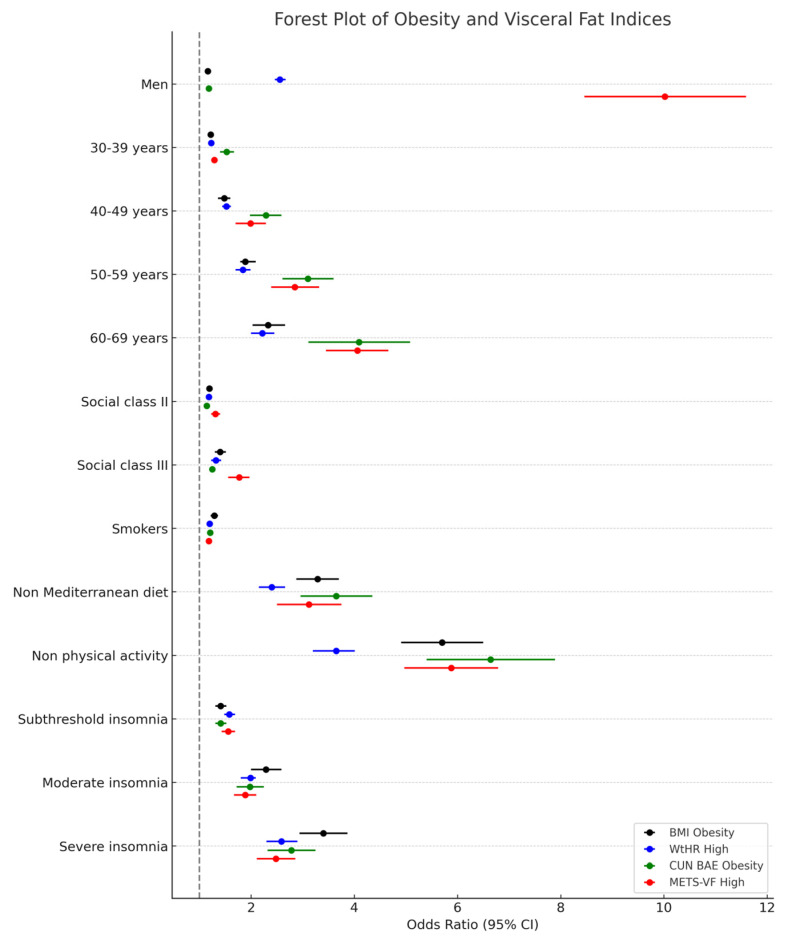
Forest Plot of Obesity and Visceral Fat Indices.

Figure 3 presents a forest plot of the associations between insomnia severity and obesity indices. BMI obesity, WtHR high, CUN-BAE obesity, and METS-VF high. Notably, METS-VF shows markedly higher ORs across all subgroups, particularly in men and older age groups, suggesting a stronger association with visceral adiposity. Lifestyle-related variables, such as physical inactivity and non-adherence to the Mediterranean diet, demonstrate the highest ORs across all indices, highlighting their critical role in metabolic health. One extreme value among male participants was retained after data validation and is presented as an outlier in Figure 3 to reflect the true range of adiposity observed. These findings underscore the importance of comprehensive lifestyle interventions in reducing obesity-related cardiometabolic risk.

## 4. Discussion

This study demonstrates that insomnia severity, unhealthy lifestyle behaviors, and sociodemographic disadvantage are independently associated with higher levels of adiposity in both men and women. These findings highlight the importance of integrating sleep assessment and behavioral health promotion into workplace health strategies.

To our knowledge, this is the first large-scale occupational study in Spain to simultaneously examine insomnia severity, lifestyle habits, and multiple validated adiposity indices within the same analytical framework. By combining both traditional (BMI, WtHR) and emerging indices (CUN-BAE, METS-VF), this work provides a multidimensional view of obesity and its determinants among employed adults.

### 4.1. Main Findings and Interpretation

In this large cross-sectional sample of Spanish workers, several consistent associations emerged. Female sex, lower physical activity, poorer adherence to the Mediterranean diet, smoking, and higher insomnia severity were all independently linked to increased adiposity. Importantly, higher insomnia scores were strongly associated with greater visceral fat accumulation, as reflected by elevated METS-VF values. These results reinforce the multifactorial nature of obesity and suggest that sleep quality represents a key, yet often overlooked, component of metabolic health in working populations.

### 4.2. Comparison with Previous Studies

Our results align with previous evidence indicating that short sleep duration and poor sleep quality are significant contributors to obesity and metabolic dysregulation [54]. For instance, meta-analyses have shown that individuals with insomnia symptoms have higher risks of obesity and cardiometabolic disturbances [55]. Similarly, large European cohorts have reported associations between insufficient sleep and increased BMI and waist circumference [56]. The strong link between insomnia and visceral adiposity observed here is consistent with the ELSA-Brasil study, which found that poor sleep quality was correlated with greater visceral fat measured by imaging techniques [57].

Physiological mechanisms may explain these associations. Sleep restriction and insomnia can disrupt leptin and ghrelin balance, elevate cortisol levels, and promote systemic inflammation—all of which contribute to increased appetite, reduced energy expenditure, and visceral fat deposition [58]. Furthermore, chronic sympathetic overactivation and altered hypothalamic–pituitary–adrenal (HPA) axis activity may exacerbate insulin resistance and metabolic imbalance in individuals with poor sleep quality [59].

In terms of lifestyle, our findings corroborate previous research showing that physical inactivity and low adherence to the Mediterranean diet are major risk factors for obesity in Mediterranean populations [60,61]. The additive role of insomnia further supports recent hypotheses suggesting a bidirectional relationship between poor sleep and unhealthy behaviors: individuals who sleep poorly are more likely to make suboptimal dietary choices and engage in sedentary lifestyles, which in turn worsen sleep patterns [62].

We also observed that women exhibited higher mean CUN-BAE and METS-VF values despite lower BMI, underscoring the need for sex-specific approaches when assessing adiposity and metabolic risk. This pattern is consistent with prior studies reporting that women may be more susceptible to sleep-related metabolic alterations, potentially mediated by hormonal and psychosocial factors [63,64].

Regarding tobacco use, our findings add to a complex body of evidence. Previous studies have shown inconsistent relationships between smoking and obesity [65], with some indicating lower body weight among smokers and others linking tobacco use to increased central adiposity [66]. In our study, smoking was independently associated with higher adiposity levels, suggesting that metabolic and behavioral factors—such as altered appetite regulation, reduced physical fitness, or compensatory dietary patterns—may contribute to this relationship among working adults.

### 4.3. Strengths and Limitations

Key strengths of this study include its large, heterogeneous occupational cohort, the use of multiple validated adiposity indices, and the standardized assessment of sleep, dietary habits, and physical activity. Incorporating both CUN-BAE and METS-VF provided a more comprehensive understanding of total and visceral fat distribution, improving the clinical interpretability of our findings.

However, several limitations must be acknowledged. The cross-sectional design prevents causal inference, and the self-reported nature of some measures (such as ISI, MEDAS-14, and IPAQ-SF) may introduce recall or social desirability bias. Residual confounding due to unmeasured factors—such as sleep apnea, chronic stress, or mental health disorders—cannot be ruled out. Additionally, because the sample consists of employed adults undergoing occupational health evaluations, the generalizability to unemployed or retired populations may be limited.

### 4.4. Implications and Future Directions

These findings underscore the need for integrated workplace interventions that simultaneously address sleep hygiene, physical activity, and dietary habits. Occupational health programs should routinely include sleep quality assessments—such as the Insomnia Severity Index—alongside metabolic screenings. Tailored interventions may be particularly beneficial for women, given their greater susceptibility to sleep-related metabolic disturbances.

Future research should employ longitudinal designs to establish causality and explore potential mediating mechanisms between sleep and obesity. Objective sleep measures (e.g., actigraphy, polysomnography) and biomarkers of stress and inflammation would further elucidate the biological pathways involved. Moreover, examining psychosocial stress, work schedules, and shift patterns could deepen understanding of how occupational factors influence the sleep–obesity relationship.

## 5. Conclusions

In summary, this study identifies insomnia severity, poor lifestyle behaviors, and sociodemographic disadvantage as independent correlates of general and visceral adiposity in Spanish workers. Poor sleep quality emerges as a key determinant of metabolic health, highlighting the need to incorporate sleep assessment into comprehensive workplace wellness programs. Interventions that jointly promote adequate sleep, healthy diet, and regular physical activity are likely to be most effective in preventing obesity and its associated comorbidities. Future longitudinal studies should confirm these associations and evaluate the impact of integrated, sex-sensitive health strategies in occupational settings.

## Figures and Tables

**Table 1:** Sociodemographic, Clinical, and Lifestyle Characteristics of the Study Population, Stratified by Sex.

	Men *n* = 51,251	Women *n* = 33,647	
Variables	Mean (SD)	Mean (SD)	*p*-Value
Age (years)	39.8 (10.3)	39.3 (10.2)	<0.001
Height (cm)	174.0 (7.0)	161.2 (6.6)	<0.001
Weight (kg)	81.1 (13.9)	65.3 (13.1)	<0.001
Waist (cm)	87.7 (9.2)	73.9 (7.9)	<0.001
Hip (cm)	100.0 (8.5)	97.2 (8.9)	<0.001
SBP (mm Hg)	124.4 (15.0)	114.3 (14.8)	<0.001
DBP (mm Hg)	75.4 (10.7)	69.6 (10.3)	<0.001
Cholesterol (mg/dL)	195.8 (38.8)	193.6 (36.5)	<0.001
HDL-c (mg/dL)	51.0 (7.0)	53.7 (7.6)	<0.001
LDL-c (mg/dL)	120.3 (37.7)	122.3 (37.1)	<0.001
Triglycerides (mg/dL)	124.1 (88.9)	88.1 (46.0)	<0.001
Glucose (mg/dL)	88.0 (12.9)	84.1 (11.4)	<0.001
Variables	*n* (%)	*n* (%)	*p*-Value
18–29 years	9129 (17.8)	6578 (19.6)	<0.001
30–39 years	17,061 (33.3)	11,039 (32.8)	
40–49 years	15,202 (29.6)	9963 (29.6)	
50–59 years	8338 (16.3)	5151 (15.3)	
60–69 years	1521 (3.0)	916 (2.7)	
Social class I	2803 (5.5)	2354 (7.0)	<0.001
Social class II	9035 (17.6)	11,230 (33.4)	
Social class III	39,413 (76.9)	20,063 (59.6)	
Smokers	19,142 (37.4)	11,029 (32.8)	<0.001
Yes Mediterranean diet	20,891 (40.8)	17,261 (51.3)	<0.001
Yes physical activity	23,221 (45.3)	17,554 (52.2)	<0.001
No insomnia	13,020 (25.4)	21,816 (64.8)	<0.001
Subthreshold insomnia	25,688 (50.1)	9260 (27.5)	
Moderate insomnia	8602 (16.8)	1904 (5.7)	
Severe insomnia	3941 (7.7)	667 (2.0)	

SBP Systolic blood pressure. DBP Diastolic blood pressure. HDL High Density Lipoprotein. LDL Low Density Lipoprotein. SD Standard deviation.

**Table 2:** Mean Values of Four Obesity and Adiposity Indices According to Sociodemographic and Lifestyle Variables, by Sex.

		BMI	WtHR	CUN BAE	METS-VF
Men	*n*	Mean (SD)	Mean (SD)	Mean (SD)	Mean (SD)
18–29 years	9129	25.0 (4.1)	0.49 (0.05)	21.0 (6.4)	5.9 (0.5)
30–39 years	17,061	26.5 (4.1)	0.51 (0.05)	24.7 (5.8)	6.3 (0.5)
40–49 years	15,202	27.4 (4.1)	0.52 (0.05)	27.1 (5.5)	6.6 (0.5)
50–59 years	8338	27.9 (4.0)	0.53 (0.05)	28.8 (5.0)	6.8 (0.5)
60–69 years	1521	28.3 (3.9)	0.53 (0.05)	30.3 (4.5)	6.9 (0.4)
Social class I	2803	26.5 (3.9)	0.50 (0.05)	25.4 (5.8)	6.3 (0.5)
Social class II	9035	26.7 (4.4)	0.51 (0.05)	25.6 (5.9)	6.4 (0.5)
Social class III	39,413	26.8 (4.3)	0.51 (0.05)	25.6 (6.4)	6.4 (0.5)
Smokers	19,142	27.1 (4.1)	0.51 (0.05)	24.6 (6.4)	6.4 (0.6)
Non smokers	32,109	26.2 (4.3)	0.51 (0.05)	26.2 (6.1)	6.3 (0.6)
Yes Mediterranean diet	20,891	24.0 (2.2)	0.48 (0.03)	21.2 (4.1)	6.1 (0.5)
Non Mediterranean diet	30,360	28.7 (4.2)	0.53 (0.05)	28.6 (5.7)	6.7 (0.5)
Yes physical activity	23,221	24.0 (2.2)	0.48 (0.03)	21.3 (4.1)	6.1 (0.5)
Non physical activity	28,030	29.1 (4.1)	0.53 (0.05)	29.2 (5.4)	6.7 (0.5)
No insomnia	13,020	23.1 (1.9)	0.47 (0.03)	19.2 (3.6)	5.9 (0.4)
Subthreshold insomnia	25,688	26.6 (2.8)	0.51 (0.04)	25.6 (4.1)	6.4 (0.4)
Moderate insomnia	8602	30.4 (4.2)	0.55 (0.05)	31.5 (4.7)	6.9 (0.3)
Severe insomnia	3941	32.4 (4.4)	0.58 (0.06)	34.2 (4.8)	7.2 (0.4)
Women	*n*	Mean (SD)	Mean (SD)	Mean (SD)	Mean (SD)
18–29 years	6578	23.9 (4.7)	0.44 (0.05)	31.4 (6.9)	5.0 (0.7)
30–39 years	11,039	24.6 (4.9)	0.45 (0.05)	33.7 (6.6)	5.3 (0.7)
40–49 years	9963	25.6 (4.8)	0.46 (0.05)	36.6 (6.0)	5.6 (0.7)
50–59 years	5151	26.7 (4.7)	0.47 (0.05)	39.2 (5.4)	5.9 (0.6)
60–69 years	916	27.3 (4.4)	0.48 (0.05)	40.9 (4.8)	6.1 (0.6)
Social class I	2354	23.7 (4.3)	0.44 (0.05)	32.8 (6.2)	5.2 (0.7)
Social class II	11,230	24.2 (4.5)	0.45 (0.05)	33.7 (6.4)	5.3 (0.8)
Social class III	20,063	25.8 (5.1)	0.47 (0.05)	36.3 (7.0)	5.6 (0.7)
Smokers	11,029	25.5 (5.0)	0.46 (0.05)	35.7 (6.9)	5.5 (0.8)
Non smokers	22,618	24.4 (4.6)	0.45 (0.05)	34.0 (6.7)	5.4 (0.7)
Yes Mediterranean diet	17,261	22.5 (2.4)	0.44 (0.04)	31.2 (4.3)	5.1 (0.6)
Non Mediterranean diet	16,386	28.0 (5.3)	0.48 (0.05)	39.3 (6.6)	5.8 (0.7)
Yes physical activity	17,554	22.3 (2.3)	0.44 (0.04)	31.0 (4.2)	5.1 (0.6)
Non physical activity	16,093	28.2 (5.1)	0.48 (0.05)	39.7 (6.2)	5.9 (0.6)
No insomnia	21,816	22.9 (2.8)	0.44 (0.04)	31.6 (4.6)	5.1 (0.6)
Subthreshold insomnia	9260	28.2 (4.5)	0.48 (0.04)	40.4 (4.8)	6.0 (0.5)
Moderate insomnia	1904	32.8 (5.4)	0.52 (0.05)	45.7 (4.8)	6.4 (0.4)
Severe insomnia	667	35.0 (5.1)	0.55 (0.06)	48.0 (4.5)	6.7 (0.4)

BMI Body Mass Index. WtHR Waist to Height Ratio. CUN BAE Clinica Universitaria de Navarra Body Adiposity Estimator. METS-VF Metabolic Score for Visceral Fat. *p* < 0.001 in all cases.

**Table 3:** Prevalence of Obesity and High Adiposity Based on Four Indices According to Sociodemographic and Lifestyle Variables, by Sex.

		BMI Obesity	WtHR High	CUN BAE Obesity	METS-VF High
Men	*n*	%	%	%	%
18–29 years	9129	10.6	31.1	22.5	2.5
30–39 years	17,061	16.9	43.9	44.1	3.6
40–49 years	15,202	22.7	53.4	63.3	11.4
50–59 years	8338	27.0	60.3	78.4	20.2
60–69 years	1521	29.0	65.9	89.5	28.1
Social class I	2803	17.3	43.6	51.6	6.8
Social class II	9035	17.9	48.9	52.1	8.0
Social class III	39,413	20.1	54.4	53.2	9.1
Smokers	19,142	21.3	50.0	56.8	8.9
Non smokers	32,109	16.6	44.1	46.2	8.6
Yes Mediterranean diet	20,891	6.2	24.5	19.1	3.9
Non Mediterranean diet	30,360	24.7	63.8	76.1	9.2
Yes physical activity	23,221	5.2	24.4	19.4	3.0
Non physical activity	28,030	27.2	67.1	80.6	12.8
No insomnia	13,020	10.8	12.1	24.6	2.9
Subthreshold insomnia	25,688	18.5	28.0	35.9	4.0
Moderate insomnia	8602	31.5	48.3	55.5	6.3
Severe insomnia	3941	39.8	58.3	66.8	8.5
Women	*n*	%	%	%	%
18–29 years	6578	10.9	11.2	25.0	0.5
30–39 years	11,039	12.7	13.5	35.6	1.2
40–49 years	9963	16.8	17.6	55.0	2.4
50–59 years	5151	21.2	22.6	77.1	5.3
60–69 years	916	24.6	25.5	89.2	8.2
Social class I	2354	8.4	10.0	31.1	2.5
Social class II	11,230	10.1	11.5	36.4	3.1
Social class III	20,063	18.8	19.2	54.9	3.8
Smokers	11,029	16.9	16.9	50.2	3.9
Non smokers	22,618	11.7	14.1	40.6	3.5
Yes Mediterranean diet	17,261	7.2	7.0	21.6	1.2
Non Mediterranean diet	16,386	20.3	29.6	74.0	4.2
Yes physical activity	17,554	6.3	5.2	18.1	1.0
Non physical activity	16,093	24.8	30.2	78.7	5.2
No insomnia	21,816	10.1	5.8	24.2	1.2
Subthreshold insomnia	9260	19.2	16.6	30.7	2.2
Moderate insomnia	1904	33.5	25.3	44.5	3.6
Severe insomnia	667	37.2	32.4	59.7	4.2

BMI Body Mass Index. WtHR Waist to Height Ratio. CUN BAE Clinica Universitaria de Navarra Body Adiposity Estimator. METS-VF Metabolic Score for Visceral Fat. SD Standard deviation. *p* < 0.001 in all cases.

**Table 4:** Adjusted Odds Ratios for Obesity and High Visceral Adiposity According to Sociodemographic, Lifestyle, and Insomnia Variables.

	BMI Obesity	WtHR High	CUN BAE Obesity	METS-VF High
	OR (95% CI)	OR (95% CI)	OR (95% CI)	OR (95% CI)
Women	1	1	1	1
Men	1.16 (1.13–1.20)	2.56 (2.46–2.67)	1.18 (1.14–1.22)	10.02 (8.46–11.59)
18–29 years	1	1	1	1
30–39 years	1.22 (1.17–1.27)	1.23 (1.19–1.28)	1.53 (1.40–1.67)	1.29 (1.24–1.34)
40–49 years	1.48 (1.36–1.60)	1.52 (1.44–1.61)	2.29 (1.98–2.59)	1.99 (1.70–2.29)
50–59 years	1.89 (1.79–2.09)	1.84 (1.70–1.99)	3.10 (2.61–3.60)	2.85 (2.39–3.32)
60–69 years	2.33 (2.03–2.66)	2.22 (2.00–2.45)	4.09 (3.11–5.08)	4.06 (3.45–4.66)
Social class I	1	1	1	1
Social class II	1.19 (1.15–1.24)	1.18 (1.14–1.22)	1.14 (1.10–1.19)	1.31 (1.23–1.40)
Social class III	1.40 (130–1.51)	1.32 (1.23–1.42)	1.25 (1.19–1.31)	1.77 (1.56–1.97)
Non smokers	1	1	1	1
Smokers	1.29 (1.22–1.36)	1.20 (1.16–1.25)	1.21 (1.16–1.27)	1.18 (1.15–1.22)
Yes Mediterranean diet	1	1	1	1
Non Mediterranean diet	3.29 (2.88–3.70)	2.40 (2.15–2.66)	3.65 (2.96–4.35)	3.12 (2.50–3.75)
Yes physical activity	1	1	1	1
Non physical activity	5.70 (4.91–6.50)	3.65 (3.20–4.01)	6.64 (5.40–7.89)	5.88 (4.97–6.79)
No insomnia	1	1	1	1
Subthreshold insomnia	1.41 (1.31–1.52)	1.58 (1.48–1.69)	1.41 (1.31–1.52)	1.56 (1.43–1.69)
Moderate insomnia	2.29 (2.00–2.59)	1.99 (1.80–2.09)	1.98 (1.72–2.25)	1.89 (1.67–2.10)
Severe insomnia	3.40 (2.94–3.87)	2.59 (2.30–2.90)	2.78 (2.32–3.25)	2.48 (2.11–2.86)

BMI Body Mass Index. WtHR Waist to Height Ratio. CUN BAE Clinica Universitaria de Navarra Body Adiposity Estimator. METS-VF Metabolic Score for Visceral Fat. OR Odds ratio. CI Confidence Interval. *p* < 0.001 in all cases.

## Data Availability

Data derived from this research are securely maintained within the facilities of ADEMA University School. The management and safeguarding of these data comply with relevant data protection laws and are overseen by the institution’s appointed Data Protection Officer, Ángel Arturo López González.
